# Impact of fertilization with reducing in nitrogen and phosphorous application on growth, yield and biomass accumulation of rice (*Oryza sativa* L.) under a dual cropping system

**DOI:** 10.7717/peerj.11668

**Published:** 2021-07-07

**Authors:** Ke Wu, Izhar Ali, Huimin Xie, Saif Ullah, Anas Iqbal, Shangqing Wei, Liang He, QianYing Huang, Xiaoyan Wu, Fangwei Cheng, Ligeng Jiang

**Affiliations:** 1 College of Agriculture, Guangxi University, Nanning, Guangxi, China; 2 Guangxi Subtropical Crops Research Institutes, Nanning, Guangxi, China; 3 College of Life Science and Technology, Guangxi University, Nanning, Guangxi, China

**Keywords:** Rice, Reducing NPK fertilization, Dry matter, Leaf Area Index, Cultivar, Yield

## Abstract

The current farming system in China is heavily reliant on synthetic fertilizers, which adversely affect soil quality and crop production. Therefore, the aim of this study was to assess the effect of different nitrogen (N) and phosphorous (P) fertilizer application rate on the growth, yield, and yield components of rice cultivars in the Binyang, Beiliu and Liucheng sites of southern China in the early (March to July) and late season (August to December). The study consisted of three fertilization regimes—CK (N_0_P_0_); N_180_P_90_ (180 kg N + 90 kg P_2_O_5_ ha^−1^) and N_90_P_45_ (90 kg N ha^−1^ + 45 kg P_2_O_5_)—conducted at each of three different experimental sites with four cultivars (Baixang 139, Y Liangyou 1, Guiyu 9, and Teyou 582). Results showed that the leaf area index (LAI) was 38.8% found higher in Guiyu 9 compared with Baxiang at reduced fertilization (N_90_P_45_). N_90_P_45_ resulted higher dry matter production at the heading (9411.2 kg ha^−1^) and maturity (15319.5 kg ha^−1^) stages in Teyou 582 at Beiliu. Fertilization (N_180_P_90_) had higher effective panicle number (4,158,800 panicle ha^−1^) and grains panicle^−1^ (113.84 grains) compared with other treatments. Teyou 582 treated with N_90_P_45_ and Y Liangyou 1 treated with N_180_P_90_ improved seed setting rate average by 82.91% and 72.17% compared with other treatments at Beiliu in both seasons, respectively. N_0_P_0_ and N_90_P_45_ increased the thousand-grain weight (TGW) of Y Liangyou 1 at Binyang (27.07 g) and Liucheng (27.84 g) during the early and late seasons, respectively. In Beiliu, the N_90_P_45_ treatment (6611.7 kg ha^−1^) of Teyou 582 increased grain yield compared with other treatments. Overall, our results suggested that reducing N and P at the ratio of 90:45 kg ha^−1^ in Teyou 582 and Y Liangyou 1 could increase rice grain yield and yield components.

## Introduction

The steady rise of fertilizer use in global agriculture over the past century has made a major contribution to the development of modern farming. It has improved overall global agricultural productivity, crop yields, and soil fertility. As a result of increased fertilizer use, agriculture production, crop yield and soil fertility were increased globally (Wehmeyer, de Guia & Connor, 2020). Since its introduction during the Green Revolution in the 1960s, synthetic fertilizer has been an essential factor of Asian agriculture (Lu & Tian, 2017). China used 26.7% of global fertilizers in 2014, resulting in China the world’s largest consumer of fertilizer (Heffer, Gruère & Roberts, 2014). A study reported that farmers in China reached an extremely high average NPK fertilizer application rate of 559.8 kg ha^−1^ (Wehmeyer, de Guia & Connor, 2020). In particular, the use of N fertilizer in rice cultivation usually overtakes 250 kg ha^−1^ (Ali et al., 2020), which is approximately higher than the global average.

The increase in the world population has led to increases in the demand for food. However, meeting this increased demand by enhancing crop production in current conventional farming systems is a major challenge. Nitrogen (N) has a significant effect on plant growth and production under various environmental conditions (Ullah et al., 2021; Leghari et al., 2016). The application of N through chemical fertilizer is the primary source of N in crop production (Tao et al., 2015; Ali et al., 2019), yet the excessive use of chemical fertilizer adversely affects soil health, the environment, and crop production (Ali et al., 2020). Furthermore, the overuse of N fertilization enhances plant growth (Iqbal et al., 2020) and decreases grain quality (Iqbal et al., 2021) and grain yield (Zörb et al., 2010). The constant increase in N fertilizer application in paddy rice production in China has led to low N use efficiency. According to the Chinese Ministry of Agriculture in 2015, the utilization rates of N, phosphorus (P), and potassium (K) fertilizers in three major cereal crops (i.e., rice, wheat, and maize) are only 33%, 24%, and 42%, respectively (Bai et al., 2016). The excessive application of N fertilizer and the low N use efficiency in paddy fields leads to a large loss of N fertilizer. The annual planting area of rice in China is 30 million hectares (Gao et al., 2014). According to Beckinghausen et al. (2020), at least 1.8 million tons of N fertilizer (pure N) are wasted every year. This is a major global challenge to feed the growing global population by increasing crop yield and quality while minimizing environmental costs. P fertilization plays a critically important role in improving rice yield. However, its utilization efficiency during agricultural consumption is low in China, which results in a serious wastage of phosphate rock resources (Li et al., 2014). Excessive fertilizer application not only leads to waste but also increases the planting costs of producers. Currently, approximately 67% of the world’s cultivated land can be found lacking in P (Dhillon et al., 2017). P deficiency in China is particularly severe, with approximately two-thirds of the cultivated land lacking P (Zhou et al., 2017a). Given that soil P deficiency is one of the main factors limiting crop yields, there is a need for more work to maximize P utilization efficiency.

Rice is the staple food of more than half of the world’s population and almost 60% of China (Zhao et al., 2020). Rice yield is mainly associated with N and P application rate (Iqbal et al., 2019), but N and P management is often modified depending on rice type, cultivar, geographic zone, and other crop practices (Angus et al., 1994; Hirzel et al., 2020). Additionally, rice yield can be induced through variety selection and improvements in cultivation technology (Cai, Liang & Wan, 2010). The selection of hybrid cultivars has a higher return compared with conventional rice (Lin et al., 2020). Furthermore, rice yield is associated with the photosynthetic products of leaves and the photosynthetic attributes rates (Acevedo-Siaca et al., 2020). Thus, increasing plant biomass is a direct way to improve rice yield, as rice cultivars with higher biomass, higher leaf area index, and high biological yield are considered more desirable.

Identifying N and P-efficient cultivars requiring minimal fertilizer application is important for improving the yield and quality of rice as well as for environmental protection (Zhou et al., 2017b). The objective of this study was to assess differences in the growth, biomass accumulation, and grain yield of cultivars under high and low N and P fertilization rates at different experimental sites.

## Materials and Methods

### Experimental sites and weather

The field experiments were conducted at three locations: (1) Binyang, Nanning, China (23°0635″N, 108°5912″E); (2) Beiliu, Yulin, China (22°4419″N, 110°1032″E); and (3) Liucheng, Liuzhou, China (24°44197″N, 109°0418″E) during the early (March to July) and late season (August to December) in 2019. The basic soil physicochemical properties of the experimental fields before the experiments are shown in Table 1.

**Table 1:** Basic physical and chemical properties of soil in the experimental site.

Site	pH	OC (g kg^−1^)	TN (g kg^−1^)	AN (mg kg^−1^)	AP (mg kg^−1^)	AK (mg kg^−1^)
Binyang	5.28	33.47	2.07	147	124.13	194
Beiliu	4.89	22.76	1.31	161	380.12	115
Liucheng	8.08	42.35	2.74	206.5	104.5	81

**Note: **

OC, organic carbon; TN, total nitrogen; AN, available nitrogen; AP, available phosphorous; AK, available potassium.

The soils of Binyang and Beiliu are relatively acidic, and the soil of Liucheng is relatively alkaline (Table 1). The content of organic carbon and available N is higher in Liucheng soil than in Binyang and Beiliu soils. The content of available P is higher in Beiliu soil compared with the soils of Binyang and Liucheng. Our previous study at the same experimental sites was conducted with the same cultivars, which show no significant effect among experimental sites for rice yield. However, their results were significant among the treatments across the experimental sites (Xie et al., 2021).

**Figure 1:**
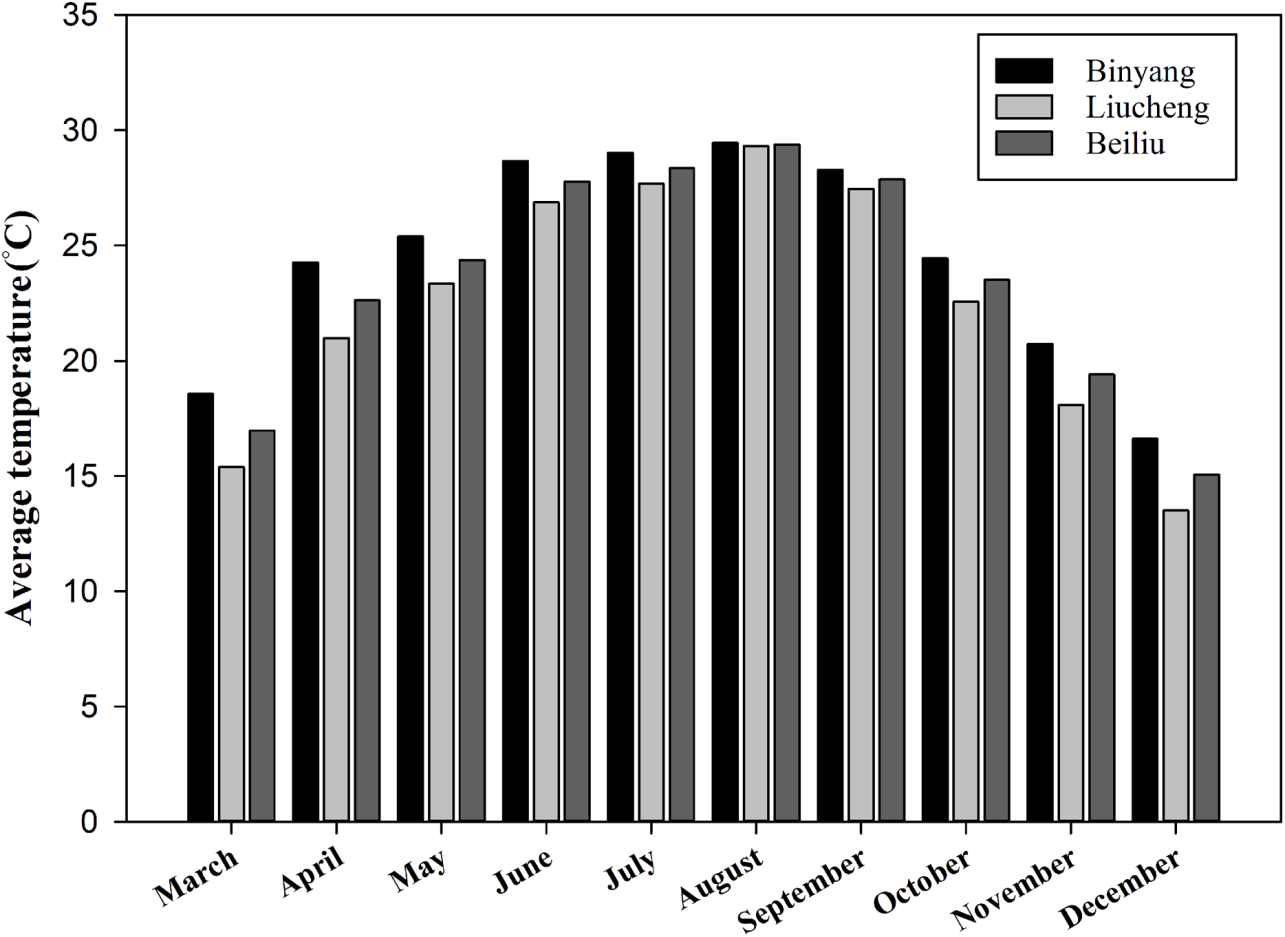
Average temperature of Binyang, Liucheng and Beiliu throughout the growing season.

**Figure 2:**
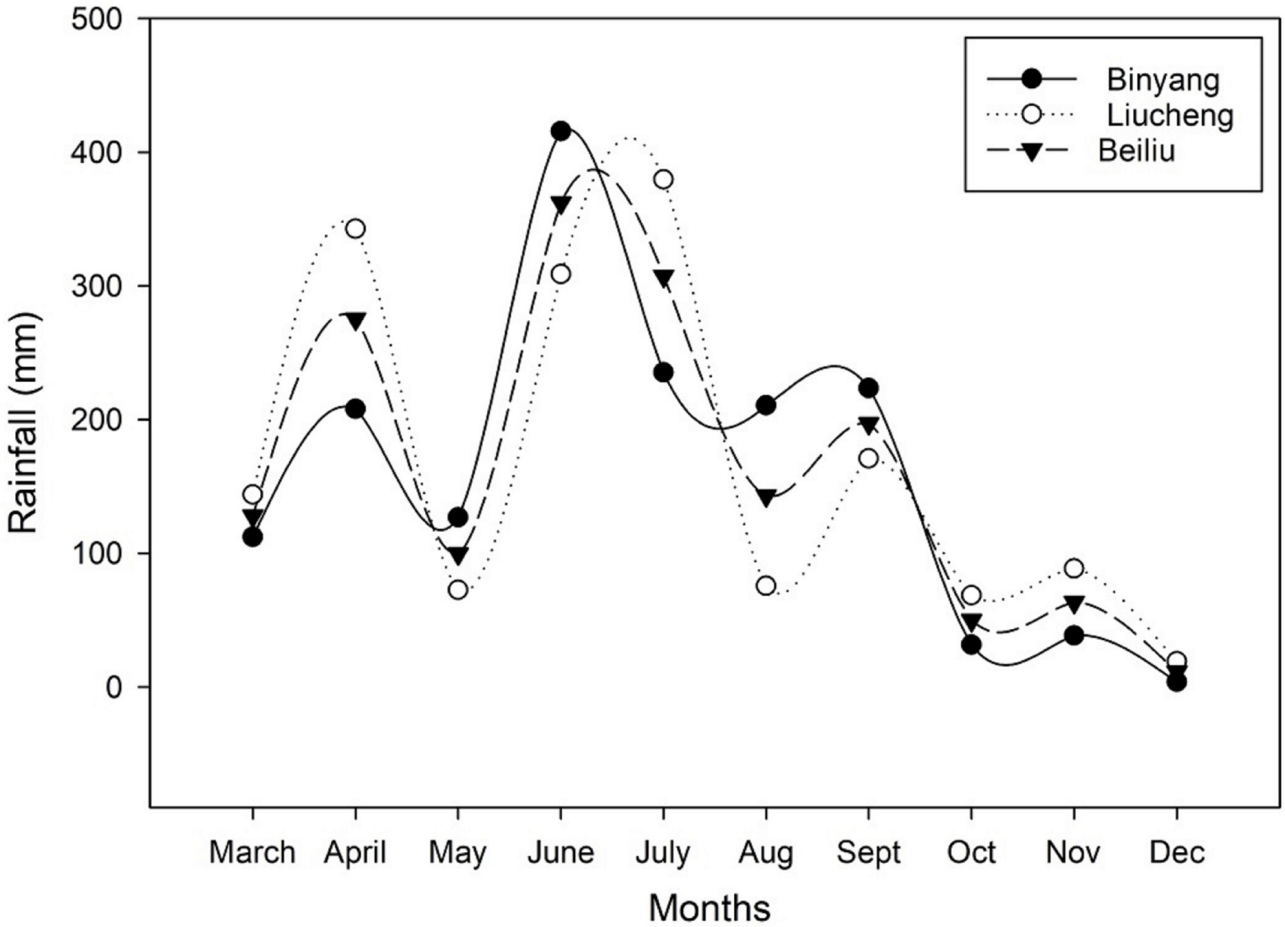
Average rain fall of Binyang, Liucheng and Beiliu throughout the growing season.

Figure 1 shows the temperature of the experimental sites collected from local metrological stations. The maximum temperature in August an average 28 °C, and the minimum temperature in December an average 10 °C. Figure 2 shows that the average rainfall of the three sites was concentrated in the early season (March to July) and late season (August to December).

### Experimental design and field management

The experiments were performed in a split-plot factorial design consisting of 12 treatments. Plots (3.6 m × 5.6 m) had an area of 20.16 m^2^. Three seedlings were planted per hill, the hill to hill distance was 14 cm, and the row-to-row distance was 30 cm. Each plot contained 12 rows, 40 hills, and 1,440 seedlings. The cultivars were randomly arranged and were not separated. Four different cultivars selected from a previous experiment (Li et al., 2019) were tested, including two hybrid rice cultivars (Teyou 582 and Y Liangyou 1) and two conventional rice cultivars (Baixiang 139 and Guiyu 9). Three fertilization levels were set for each cultivar: (1) F1: 180 kg N ha^−1^ + 90 kg P_2_O_5_ ha^−1^ + 180 kg K_2_O ha^−1^ (N_180_P_90_), (2) F2: 90 kg N ha^−1^ + 45 kg P_2_O_5_ ha^−1^ + 180 kg K_2_O ha^−1^ (N_90_P_45_), and (3) F3: 0 kg N ha^−1^ + 0 kg P_2_O_5_ ha^−1^ + 180 kg K_2_O ha^−1^ (N_0_P_0_).

The 25-day-old seedlings were manually transplanted to the experimental fields. The sources of N, P, and K were urea, superphosphate, and potassium chloride, respectively. Urea and potassium chloride were applied in splits (50% as a basal fertilizer, 30% at the tillering stage, and 20% at the panicle initiation stage), whereas all superphosphate was used as basal fertilizer. Uniform flood water approximately 4 cm deep was continued from transplanting until physiological maturity in each plot. Throughout the growing season, standard agricultural practices including irrigation, herbicide application, and insecticide application were performed in the same manner for all plots.

### Sampling and measurements

#### Soil sampling and analysis

Prior to experiments, soil samples were randomly collected from up to 20 cm depth, air-dried, and passed through a 2-mm sieve. Soil pH was assessed after shaking the soil with distilled water at a 1:2.5 (w/v) solid-to-water ratio for 1 h using a digital pH meter (Thunderbolt PHS-3C, Shangai, China) (Cambardella et al., 2001). Total organic carbon was measured following the procedure of (Rich & Black, 1994). Total nitrogen (TN) was analyzed using the micro-Kjeldahl procedure (Jackson, 1956). Next, 200-mg soil samples were digested using the salicylic acid–sulfuric acid–hydrogen peroxide method (Ohyma et al., 1991), and available nitrogen (AN) was extracted from the soil samples using the hot water extraction method (Curtin et al., 2006). Available phosphorous (AP) was determined by Olsen’s method with 0.5 M NaHCO_3_ solution adjusted to pH 8.5 (Olsen, 1964). Available K was determined by placing the soil samples in 100-mL polyethylene bottles and adding 50 mL of the ammonium acetate/acetic acid solution (AK). AP was determined by the method as previously described by Leaf (1958).

#### Leaf area index

During the heading and maturity stages of rice, five representative rice samples were taken from each treatment plot, prepared for preservation, and brought to the laboratory to determine the rice leaf area using the length-width coefficient method (Xie et al., 2021).

#### Dry matter accumulation

To measure dry matter accumulation (DM), samples were randomly collected at the heading and maturity stages. The above-ground plants were washed with water and divided into three parts—stem, leaf, and panicle—and were dried in an oven at 70 °C for 48 h. Finally, the samples were weighed with a digital lab scale.

#### Growth, yield, and yield components

Five rice tillers were selected randomly to determine the number of filled grains per panicle, the number of grains blighted, seed setting rate, and thousand-grain weight (TGW). All of the plants in the plot were harvested, threshed, dried, and weighed. The moisture content of 100-g grains was measured and converted into standard yield based on 15% water content.

#### Statistical analysis

The data were analyzed using Statistix 8.0 software (Miller Landing Rd Tallahassee, FL 32312). After checking the data for normality, data were analyzed using two-way ANOVAs. The least significant difference (LSD) tests at (*P* < 0.05) were performed to assess significant differences among treatments (Steel, Torrie & Dickey, 1996).

## Results

### Significant and non signifant data

The significant level of rice traits as influenced by experimental sites (E), treatments (T), varieties (V), E × T, E × V and E × T × V is shown in Table 2. All interactions were found non-significant, except E × T × V for grain yield. Among the treatments, rice grain yield, grains panicle^−1^ and Seed setting rate were found non-significant during the early season, whereas during late season seed setting rate and thousand-grain weight were recorded non-significant. In the case of different varieties (V) all data were found significant during the early season and during late season, leaf area, dry matter and grain yield were found non-significant (Table 2).

**Table 2:** Analysis of variance for leaf area, dry matter, grain yield, effective panicle numbers, grains panicle^−1^, seed setting rate, thousand-grain weight, as affected by experimental sites (E), treatments (T) and varieties (V) early and late sowing sea.

Rice traits	Experimental sites (E)	Treatments(T)	Varieties (V)	ExT	ExV	TxV
**Early Season**						
Leaf area	**	*	*	*	*	ns
Dry matter	**	*	*	ns	*	ns
Grain yield	**	ns	**	**	**	ns
Effective panicle numbers	**	**	**	ns	**	ns
Grains panicle^−1^	**	ns	**	ns	*	ns
Seed setting rate	ns	ns	*	ns	**	ns
Thousand-grain weight	**	ns	**	ns	ns	ns
**Late season**						ns
Leaf area	**	**	ns	ns	ns	ns
Dry matter	**	**	ns	ns	*	ns
Grain yield	**	**	ns	**	**	ns
Effective panicle numbers	**	*	**	ns	ns	ns
Grains panicle^−1^	**	**	*	ns	**	ns
Seed setting rate	**	ns	*	ns	ns	ns
Thousand-grain weight	ns	ns	**	ns	**	ns

**Note: **

ns stands for non-significant, while *, **, and *** stand for significance at the 5, 1, and 0.1% levels of probability.

### Effects of *N* and *P* levels on leaf area index

The leaf area index (LAI) of rice was significantly affected by fertilizer rate, experimental sites, and cultivar during both seasons (Table 3). LAI was 21.5% and 52.1% higher in Binyang and Beiliu, respectively, across both seasons compared with Liucheng. The LAI was highest for Guiyu 9 during the early season, which was 38.8% higher compared with Baixiang 139; during the late season, the LAI was highest for Teyou 582, which was 17.8% higher compared with Guiyu 9. Y Liangyou 1 under N_180_P_90_ resulted in higher LAI (3.85 m^−2^) across seasons compared with the rest of the treatments. However, the lowest value of LAI (0.85 m^−2^) was observed for Y Liangyou 1 at Binyang under N_0_P_0_ treatment.

**Table 3:** Leaf area index (LAI) at ripening stage of different rice cultivars under different nitrogen and phosphorus rate in the early season of 2019.

Season	Site	Treatment	Baixiang139	Guiyu 9	Y Liangyou 1	Teyou 582	Average
Early		N_180_P_90_	3.00 ± 0.99abc	3.85 ± 0.18a	2.75 ± 0.72abc	2.66 ± 0.63bc	3.07
	Binyang	N_90_P_45_	2.71 ± 0.81bc	3.38 ± 0.12ab	2.42 ± 0.14bc	2.95 ± 0.94abc	2.87
		N_0_P_0_	2.32 ± 0.64bc	2.83 ± 0.43abc	2.12 ± 0.42c	2.25 ± 0.22c	2.38
		Average	2.68	3.35	2.43	2.62	2.77
		N_180_P_90_	2.37 ± 0.58abc	3.61 ± 0.62a	2.40 ± 0.62abc	2.52 ± 0.78abc	2.73
	Beiliu	N_90_P_45_	1.84 ± 0.51bc	3.25 ± 0.49ab	2.17 ± 1.20abc	1.87 ± 0.16bc	2.28
		N_0_P_0_	1.26 ± 0.35c	2.46 ± 0.95abc	1.97 ± 0.29abc	1.58 ± 0.28c	1.82
		Average	1.82	3.11	2.18	1.99	2.28
		N_180_P_90_	2.39 ± 0.34ab	3.38 ± 0.53ab	3.85 ± 1.53a	3.14 ± 1.25ab	3.19
	Liucheng	N_90_P_45_	2.12 ± 0.90ab	2.65 ± 0.79ab	2.70 ± 0.86ab	2.18 ± 0.29ab	2.41
		N_0_P_0_	1.56 ± 0.29b	1.75 ± 0.20ab	1.20 ± 0.33b	1.78 ± 0.58ab	1.57
		Average	2.02	2.59	2.58	2.37	2.39
Late		N_180_P_90_	2.63 ± 0.30ab	1.12 ± 0.49cd	2.24 ± 1.04abc	2.97 ± 0.49a	2.24
	Binyang	N_90_P_45_	1.89 ± 1.80abcd	1.42 ± 0.81bcd	1.36 ± 0.09bcd	1.61 ± 1.01bcd	1.57
		N_0_P_0_	1.41 ± 0.42bcd	0.97 ± 0.16cd	0.85 ± 0.13d	1.36 ± 0.28bcd	1.15
		Average	1.98	1.17	1.48	1.98	1.65
		N_180_P_90_	3.12 ± 0.25ab	2.96 ± 0.67ab	3.30 ± 1.03a	3.07 ± 0.74ab	3.11
	Beiliu	N_90_P_45_	2.47 ± 0.27abc	2.56 ± 1.08abc	2.39 ± 0.53abc	3.16 ± 1.05ab	2.65
		N_0_P_0_	2.03 ± 0.21abc	1.75 ± 0.49bc	1.36 ± 0.43c	1.90 ± 0.46abc	1.76
		Average	2.54	2.42	2.35	2.71	2.51
		N_180_P_90_	2.64 ± 0.44ab	3.16 ± 0.35a	2.78 ± 0.49ab	2.40 ± 0.82abc	2.75
	Liucheng	N_90_P_45_	1.93 ± 0.20bcd	1.96 ± 0.96bcd	1.98 ± 0.08bcd	2.42 ± 0.63abc	2.07
		N_0_P_0_	1.39 ± 0.23d	1.49 ± 0.26d	1.61 ± 0.33cd	1.59 ± 0.21cd	1.52
		Average	1.99	2.2	2.12	2.14	2.11

**Note: **

Values in columns with different letters showed significant differences (*P* < 0.05). ± Value represents the SE value among the replications.

### Effects of *N* and *P* levels on DM

In the early season, N and P fertilizers, site, and cultivar significantly affected DM during both seasons and regimes (Tables 4 and 5). The highest DM was observed at Liucheng and Beiliu, respectively, which had 23.5% and 11.7% higher DM compared withBeiliu. Y Liangyou 1 resulted in 20.8% and 21.2% higher DM compared with Baixiang 139 at the heading and maturity stages, respectively.

**Table 4:** Dry matter accumulation at heading and maturity stage of different rice cultivars under different nitrogen and phosphorus rate in the early season of 2019 (kg/ha).

Site	Treatment	Baixiang139	Guiyu 9	Y Liangyou 1	Teyou 582	Average
		**Heading**				
** **	N_180_P_90_	6,697.1 ± 403.93abc	6,888.6 ± 386.41abc	8,055.0 ± 986.89ab	8,541.9 ± 855.01a	7,545.7
Binyang	N_90_P_45_	6,360.5 ± 900.03abc	7,584.9 ± 1,127.33ab	8,051.0 ± 1,260.84ab	7,777.7 ± 512.36ab	7,443.5
	N_0_P_0_	4,948.1 ± 1,725.84c	6,117.8 ± 368.12bc	5,949.5 ± 574.96bc	7,280.3 ± 538.57ab	6,073.9
	Average	6,001.9	6,863.8	7,351.8	7,866.6	7,021
	N_180_P_90_	8,069.3 ± 1,280.14ab	10,228.5 ± 1,520.39a	8,925.3 ± 970.03a	8,710.4 ± 147.70ab	8,983.4
Beiliu	N_90_P_45_	8,660.4 ± 1,502.70ab	9,158.9 ± 759.78a	7,923.9 ± 1,201.95ab	9,411.2 ± 1,088.72a	8,788.6
	N_0_P_0_	4,942.7 ± 217.27b	7,701.6 ± 1,371.37ab	7,140.3 ± 783.79ab	7,222.8 ± 184.64ab	6,751.9
	Average	7,224.1	9,029.7	7,996.5	8,448.1	8,174.6
	N_180_P_90_	6,367.8 ± 745.71ab	7,467.0 ± 1,093.67ab	8,109.8 ± 1,340.07a	6,471.5 ± 2,637.84ab	7,104
Liucheng	N_90_P_45_	6,194.9 ± 1,013.22ab	6,937.2 ± 603.47ab	8,369.0 ± 513.12a	6,946.4 ± 71.40ab	7,111.9
	N_0_P_0_	4,424.4 ± 864.94b	6,289.4 ± 975.70ab	5,945.0 ± 795.21ab	5,883.0 ± 748.81ab	5,635.5
	Average	5,662.4	6,897.9	7,474.6	6,433.6	6,617.1
		**Maturity**				
** **	N_180_P_90_	9,752.1 ± 760.60bc	10,694.0 ± 712.45abc	14,656.8 ± 1,973.58a	12,380.1 ± 1,746.95ab	11,870.8
Binyang	N_90_P_45_	9,707.7 ± 1,305.80bc	11,627.9 ± 77.92abc	13,238.9 ± 301.60ab	13,948.5 ± 3,501.99a	12,130.8
	N_0_P_0_	8,323.4 ± 1,305.58c	9,735.8 ± 371.20bc	11,190.9 ± 1,874.89abc	10,950.9 ± 538.05abc	10,050.3
	Average	9,261.1	10,685.9	13,028.9	12,426.5	11,350.6
	N_180_P_90_	14,076.9 ± 2,167.13ab	12,074.9 ± 1,237.51ab	14,144.1 ± 2,887.99ab	13,709.1 ± 1,391.80ab	13,501.3
Beiliu	N_90_P_45_	13,116.5 ± 3,666.66ab	11,549.3 ± 2,110.37ab	15,319.5 ± 1,724.47a	13,655.0 ± 1,257.55ab	13,410.1
	N_0_P_0_	9,356.4 ± 1,084.59b	10,661.9 ± 2,503.85ab	12,306.8 ± 1,875.56b	12,212.4 ± 2,576.85ab	11,134.4
	Average	12,183.3	11,428.7	13,923.5	13,192.2	12,681.9
	N_180_P_90_	11,717.1 ± 339.72abcd	12,961.8 ± 1,772.77abc	14,030.4 ± 1,539.90ab	14,958.0 ± 1,137.26a	13,416.8
Liucheng	N_90_P_45_	10,587.0 ± 1,710.58abcd	12,929.3 ± 1,700.94abc	12,189.2 ± 1,372.23abcd	12,437.0 ± 923.96abcd	12,035.6
	N_0_P_0_	8,496.6 ± 2,145.74cd	9,628.7 ± 2,049.71bcd	8,253.8 ± 1,621.48d	10,361.0 ± 2,802.33bcd	9,185
	Average	10,266.9	11,839.9	11,491.1	12,585.3	11,545.8

**Note: **

Values in columns with different letters showed significant differences (*P* < 0.05).

**Table 5:** Dry matter accumulation at heading and maturity stage of different rice cultivars under different nitrogen and phosphorus rate in the late season of 2019 (kg ha^−1^).

Site	Treatment	Baixiang139	Guiyu 9	Y Liangyou 1	Teyou 582	Average
	**Heading**					
	N_180_P_90_	9,686.6 ± 741.43ab	10,083.6 ± 955.38a	8,549.0 ± 1,741.78abcd	8,936.7 ± 1,678.65abc	9,314
Binyang	N_90_P_45_	8,323.7 ± 1,235.71abcd	7,611.9 ± 92.63abcde	6,798.6 ± 1,063.82bcde	9,067.8 ± 1,465.35abc	7,950.5
	N_0_P_0_	5,498.1 ± 1,852.39de	6,083.0 ± 1,192.46cde	4,362.2 ± 468.44e	6,779.3 ± 2,028.19bcde	5,680.7
	Average	7,836.1	7,926.2	6,569.9	8,261.3	7,648.4
	N_180_P_90_	8,043.2 ± 2,367.80a	7,131.8 ± 418.48ab	7,910.0 ± 1,069.79a	7,913.7 ± 720.96a	7,749.7
Beiliu	N_90_P_45_	7,280.7 ± 762.72ab	7,215 ± 831.09ab	7,186.4 ± 83.32ab	7,427.9 ± 936.24ab	7,277.5
	N_0_P_0_	5,069.4 ± 291.30b	5,706.5 ± 637.24ab	6,271.5 ± 369.49ab	5,836.8 ± 1,025.77ab	5,721.1
	Average	6,797.8	6,684.4	7,122.6	7,059.5	6,916.1
	N_180_P_90_	9,364.8 ± 962.94ab	10,149.6 ± 296.78a	9,913.4 ± 567.71ab	9,693.2 ± 626.09ab	9,780.3
Liucheng	N_90_P_45_	7,658.4 ± 1,957.01ab	9,905.1 ± 1,565.78ab	9,691.8 ± 385.80ab	9,447.6 ± 873.91ab	9,175.7
	N_0_P_0_	7,574.7 ± 726.67ab	7,327.8 ± 555.09b	8,510.7 ± 485.66ab	7,537.1 ± 1,164.75ab	7,737.6
	Average	8,199.3	9,127.5	9,372	8,892.6	8,897.9
	**Maturity**					
	N_180_P_90_	11,818.8 ± 2,372.51ab	9,754.1 ± 750.08abcd	11,454.3 ± 1,363.42ab	12,077.1 ± 3,098.66a	11,276.1
Binyang	N_90_P_45_	8,922.5 ± 1,348.79abcde	9,304.5 ± 358.40abcde	8,993.4 ± 585.10abcde	10,953.5 ± 2,187.7abc	9,543.5
	N_0_P_0_	6,789.5 ± 1,366.36de	7,367.7 ± 856.38cde	5,617.5 ± 274.53e	8,122.4 ± 1,714.71bcde	6,974.3
	Average	9,176.9	8,808.8	8,688.4	10,384.3	9,264.6
	N_180_P_90_	12,020.7 ± 557.22ab	11,372.7 ± 1,048.47abc	13,421.7 ± 1,356.26a	10,044.3 ± 839.54abcd	11,714.9
Beiliu	N_90_P_45_	10,467.9 ± 1,468.12abcd	9,393.2 ± 2,763.04bcd	10,796.1 ± 287.97abcd	12,324.6 ± 1,343.67ab	10,745.4
	N_0_P_0_	8,660.9 ± 531.44bcd	7,445.4 ± 1,992.97d	7,758.9 ± 2,549.71cd	7,197.45 ± 1,338.98d	7,765.7
	Average	10,383.2	9,403.8	10,658.9	9,855.5	10,075.3
	N_180_P_90_	11,552.4 ± 1,724.73abc	11,865.2 ± 1,080.48abc	13,414.1 ± 2,210.51a	12,579.8 ± 1,813.34ab	12,352.9
Liucheng	N_90_P_45_	9,900.2 ± 609.73abc	9,977.3 ± 1,427.54abc	12,352.2 ± 862.57abc	11,979.9 ± 1,079.22abc	11,052.4
	N_0_P_0_	9,110.1 ± 1,233.77bc	8,817.8 ± 700.49c	11,366.9 ± 782.50abc	10,807.1 ± 1,033.32abc	10,025.5
	Average	10,187.6	10,220.1	12,377.7	11,788.9	11,143.6

**Note: **

Values in columns with different letters showed significant differences (*P* < 0.05).

The highest values of DM at the heading (9,411.2 kg ha^−1^) stages were recorded for Teyou 582 treated with N_90_P_45_ at Beiliu. However, the lowest DM at the heading (4,424.4 kg ha^−1^) and maturity (8,253.8 kg ha^−1^) stages were recorded for Baixiang 139 and Y Liangyou 1 under N_0_P_0_ at Liucheng during the early season.

During the heading stage of late rice, the DM of Liucheng among different treatments was 28.7% higher than that of Beiliu, and the DM of Liucheng among different treatments during the maturity period was 20.3% higher than that of Binyang (Table 5). Teyou 582 had 6% and 12.6% higher DM in the late-season compared with Baixiang 139 and Guiyu 9 at the heading and maturity stages, respectively. N_180_P_90_ led to higher DM (10,083.6 kg ha^−1^ and 13,421.7 kg ha^−1^) in Guiyu 9 and Y Liangyou 1 at Binyang and Beiliu, respectively, compared with the other cultivars and experimental sites. However, the lowest DM (4,362.2 kg ha^−1^ and 5,617.5 kg ha^−1^) during the late season was observed for Y Liangyou 1 at Binyang under N_0_P_0_ treatment.

### Yield and yield components

#### Effective panicle numbers

Lower rates of *N* and *P* fertilization significantly improved crop growth, grain yield, and yield components during both seasons (Tables 6 and 7). During the early rice period, the effective panicle number of Beiliu between different treatments was 28.6% higher than that of Liucheng. Compared with Teyou 582, the effective panicle number of Baixiang 139 increased by 40.3%. Furthermore, Baixiang 139 treated with N_180_P_90_ led to a higher panicle number (4,158,800 panicle ha^−1^) compared with the other treatments in Beiliu. Teyou 582 had the lowest number of panicles (1.6508 million panicle ha^−1^) under N_90_P_45_ treatment at Liucheng.

**Table 6:** Effects of N and P application rates on yield components in early season in different rice cultivars.

Site	Cultivars	Treatments	Effective	Grain	Seed setting	TGW	Grain yield
			panicles	panicle^−1^	rate (%)	(g)	(kg ha^−1^)
Binyang	Baixiang 139	N_180_P_90_	300.99 ± 14.52a	125.47 ± 4.88cd	73.9 ± 0.01ab	18.36 ± 0.22d	5,153.6 ± 135.15e
		N_90_P_45_	312.08 ± 10.98a	115.25 ± 7.86d	81.11 ± 0.08a	18.70 ± 0.68d	5,015.1 ± 56.03e
		N_0_P_0_	256.63 ± 47.52ab	125.64 ± 3.07bcd	78.88 ± 0.04ab	18.14 ± 0.06d	4,900.7 ± 83.57e
	Guiyu 9	N_180_P_90_	270.89 ± 17.14a	132.97 ± 14.54bcd	56.73 ± 0.05c	22.54 ± 0.94c	5,130.5 ± 169.89e
		N_90_P_45_	267.72 ± 2.74ab	150.48 ± 2.97abcd	55.47 ± 0.05c	22.96 ± 0.67c	5,020.4 ± 206.09e
		N_0_P_0_	256.63 ± 4.75ab	121.74 ± 6.81d	65.60 ± 0.01bc	22.02 ± 0.97c	4,318.1 ± 126.76f
	Y Liangyou 1	N_180_P_90_	281.98 ± 26.17a	133.13 ± 13.95bcd	80.93 ± 0.06a	27.00 ± 0.93a	6,333.2 ± 100.02bc
		N_90_P_45_	251.88 ± 45.34ab	154.08 ± 46.56abcd	81.58 ± 0.11a	26.48 ± 0.76a	7,231.1 ± 163.02a
		N_0_P_0_	242.38 ± 20.72ab	129.11 ± 12.16bcd	78.52 ± 0.09ab	27.07 ± 0.21a	6,635.3 ± 300.98b
	Teyou 582	N_180_P_90_	250.3 ± 21.43ab	172.22 ± 11.78abc	72.30 ± 0.09ab	24.74 ± 1.07b	6,258.0 ± 287.70c
		N_90_P_45_	251.88 ± 28.91ab	187.86 ± 30.59a	77.47 ± 0.02ab	24.73 ± 0.85b	5,794.1 ± 56.80d
		N_0_P_0_	199.6 ± 12.57b	174.66 ± 9.22ab	77.91 ± 0.06ab	25.85 ± 0.27ab	5,641.8 ± 215.56d
Beiliu	Baixiang 139	N_180_P_90_	415.88 ± 30.61a	120.52 ± 15.15c	81.08 ± 0.03a	17.62 ± 0.48d	5,125.4 ± 960.29bcd
		N_90_P_45_	361.91 ± 43.64ab	133.77 ± 19.06c	80.18 ± 0.03a	17.82 ± 0.18d	5,723.4 ± 307.21abc
		N_0_P_0_	287.3 ± 11.00bc	120.36 ± 13.32c	80.02 ± 0.07a	17.57 ± 0.22d	5,179.5 ± 254.63bcd
	Guiyu 9	N_180_P_90_	304.76 ± 33.33bc	113.84 ± 10.48c	53.61 ± 0.03bc	22.34 ± 1.95c	4,763.7 ± 212.57cd
		N_90_P_45_	282.54 ± 2.75c	119.83 ± 10.88c	45.55 ± 0.13c	21.47 ± 1.46c	4,605.9 ± 216.53d
		N_0_P_0_	277.78 ± 58.77c	115.3 ± 21.09c	49.54 ± 0.17c	21.75 ± 0.31c	4,799.0 ± 167.32bcd
	Y Liangyou 1	N_180_P_90_	273.02 ± 11.98c	138.01 ± 24.85c	74.34 ± 0.08a	25.83 ± 0.75ab	5,843.3 ± 138.81ab
		N_90_P_45_	261.91 ± 19.05c	159.62 ± 22.51abc	82.29 ± 0.01a	26.06 ± 0.73ab	6,546.8 ± 64.57a
		N_0_P_0_	233.33 ± 16.50cd	143.82 ± 15.93bc	70.86 ± 0.04ab	26.95 ± 0.46a	6,361.8 ± 432.49a
	Teyou 582	N_180_P_90_	253.97 ± 29.10c	194.53 ± 25.84a	74.39 ± 0.11a	22.30 ± 0.63c	6,377.3 ± 286.02a
		N_90_P_45_	231.75 ± 21.99cd	186.71 ± 30.66ab	82.91 ± 0.10a	23.87 ± 1.47bc	6,611.7 ± 196.59a
		N_0_P_0_	174.60 ± 58.19d	200.89 ± 4.37a	80.81 ± 0.09a	25.15 ± 0.65ab	6,545.9 ± 285.52a
Liucheng	Baixiang 139	N_180_P_90_	293.65 ± 11.00a	139.05 ± 1.85bc	74.86 ± 0.08abc	17.39 ± 0.44e	4,305.6 ± 120.33bcd
		N_90_P_45_	269.84 ± 11.98ab	142.15 ± 19.57bc	75.93 ± 0.04abc	17.12 ± 0.33e	4,474.2 ± 676.31abc
		N_0_P_0_	209.52 ± 31.23cde	140.94 ± 11.65bc	82.4 ± 0.05a	17.05 ± 0.79e	3,482.3 ± 214.63d
	Guiyu 9	N_180_P_90_	241.27 ± 11.98abc	154.18 ± 31.29bc	66.09 ± 0.03bc	21.12 ± 0.83d	4,137.0 ± 310.68cd
		N_90_P_45_	212.70 ± 13.75bcde	187.91 ± 17.81ab	69.25 ± 0.03abc	20.89 ± 0.53d	4,166.7 ± 467.74cd
		N_0_P_0_	201.59 ± 13.75cde	148.17 ± 18.52bc	66.11 ± 0.09bc	20.76 ± 0.42d	3,740.1 ± 274.88cd
	Y Liangyou 1	N_180_P_90_	228.57 ± 16.50bcd	163.91 ± 15.73bc	63.53 ± 0.08c	25.29 ± 0.37a	5,198.4 ± 61.91ab
		N_90_P_45_	212.7 ± 11.98bcde	160.49 ± 9.62bc	66.37 ± 0.11bc	25.58 ± 0.66a	5,238.2 ± 337.98ab
		N_0_P_0_	173.02 ± 2.75de	132.53 ± 23.66c	73.57 ± 0.04abc	24.49 ± 0.86ab	4,414.8 ± 474.65abcd
	Teyou 582	N_180_P_90_	225.40 ± 45.01bcd	215.94 ± 43.82a	80.16 ± 0.03ab	22.93 ± 0.25c	5,287.8 ± 574.25a
		N_90_P_45_	165.08 ± 14.55e	228.4 ± 28.65a	82.41 ± 0.04a	22.98 ± 0.23c	4,613.1 ± 658.10abc
		N_0_P_0_	177.78 ± 32.41de	177.28 ± 18.54abc	81.55 ± 0.04a	23.48 ± 0.25bc	4,652.9 ± 725.56abc

**Note: **

Values in columns with different letters showed significant differences (*P* < 0.05).

**Table 7:** Effects of *N* and *P* application rates on yield components of late rice in different rice cultivars.

Site	Cultivars	Treatments	Effective	Total grains	Seed setting	TGW(g)	Actual output
			panicles	per panicle	rate (%)		(kg ha^−1^)
Binyang	Baixiang 139	N_180_P_90_	358.02 ± 42.60a	127.46 ± 13.76de	42.51 ± 0.07bc	18.29 ± 0.62c	2,980.5 ± 611.19abcd
		N_90_P_45_	266.14 ± 41.43b	135.35 ± 8.44cde	56.56 ± 0.03ab	19.39 ± 0.72c	3,588.3 ± 196.74a
		N_0_P_0_	237.62 ± 49.62bc	108.11 ± 2.68ef	58.89 ± 0.03a	18.22 ± 0.27c	1,941.9 ± 235.31e
	Guiyu 9	N_180_P_90_	185.35 ± 20.72bcd	179.85 ± 12.97a	45.48 ± 0.07abc	22.84 ± 0.78b	2,423.3 ± 314.67cde
		N_90_P_45_	199.60 ± 12.57bcd	163.09 ± 18.72abc	40.32 ± 0.04c	22.54 ± 1.41b	2,659.7 ± 373.08cde
		N_0_P_0_	148.91 ± 23.44d	169.73 ± 9.45ab	45.48 ± 0.08abc	22.08 ± 0.76b	2,254.4 ± 329.29de
	Y Liangyou 1	N_180_P_90_	237.62 ± 28.91bc	144.4 ± 6.94bcd	48.50 ± 0.08abc	26.01 ± 2.01a	3,453.3 ± 345.73ab
		N_90_P_45_	217.03 ± 15.28bcd	121.18 ± 0.95def	51.57 ± 0.01abc	25.64 ± 0.34a	3,318.2 ± 140.99ab
		N_0_P_0_	169.51 ± 2.74cd	94.79 ± 2.28f	55.83 ± 0.07ab	25.2 ± 0.53a	2,338.8 ± 168.62cde
	Teyou 582	N_180_P_90_	201.19 ± 48.08bcd	169.47 ± 5.71ab	53.86 ± 0.08abc	25.36 ± 0.29a	3,174.6 ± 373.46abc
		N_90_P_45_	202.77 ± 38.12bcd	160.91 ± 8.82abc	56.55 ± 0.02ab	25.43 ± 0.48a	3,065.0 ± 292.12abcd
		N_0_P_0_	177.43 ± 38.41cd	135.42 ± 16.04cde	51.64 ± 0.04abc	25.17 ± 0.45a	2,794.7 ± 402.43abcd
Beiliu	Baixiang 139	N_180_P_90_	292.06 ± 32.41a	142.67 ± 17.66bc	65.95 ± 0.02abc	18.81 ± 0.21e	5,537.1 ± 138.45bc
		N_90_P_45_	292.06 ± 50.92a	127.88 ± 15.63c	70.95 ± 0.03ab	18.23 ± 0.13e	4,725 ± 175.89d
		N_0_P_0_	261.91 ± 21.82ab	121.83 ± 8.16c	71.30 ± 0.04a	17.50 ± 0.07e	4,005.2 ± 57.62ef
	Guiyu 9	N_180_P_90_	192.06 ± 11.00bcd	188.24 ± 13.79ab	57.39 ± 0.06d	23.42 ± 0.79cd	4,974.2 ± 166.89cd
		N_90_P_45_	188.89 ± 49.56bcd	154.21 ± 19.85abc	60.58 ± 0.07abc	22.52 ± 1.47d	4,365.2 ± 476.59de
		N_0_P_0_	141.27 ± 27.08d	162.36 ± 24.28abc	58.05 ± 0.04d	22.73 ± 0.84d	3,590 ± 136.54f
	Y Liangyou 1	N_180_P_90_	228.57 ± 34.34abc	150.19 ± 27.02bc	72.17 ± 0.21a	26.99 ± 0.57a	6,284.7 ± 126.86a
		N_90_P_45_	204.76 ± 12.60bcd	145.69 ± 7.03bc	71.31 ± 0.06a	26.01 ± 0.96ab	6,063.2 ± 628.91ab
		N_0_P_0_	155.56 ± 43.21cd	136.51 ± 12.52c	68.53 ± 0.04abc	26.78 ± 0.51a	4,623.5 ± 413.43de
	Teyou 582	N_180_P_90_	176.19 ± 4.76cd	164.21 ± 8.90abc	59.45 ± 0.07bc	26.42 ± 0.31ab	5,795.6 ± 265.96ab
		N_90_P_45_	179.37 ± 2.75cd	199.60 ± 31.25a	66.41 ± 0.04abc	26.40 ± 0.18ab	5,435.6 ± 680.58bc
		N_0_P_0_	144.45 ± 7.27d	153.27 ± 23.63abc	66.08 ± 0.07abc	24.85 ± 0.87bc	4,466.6 ± 130.83de
Liucheng	Baixiang 139	N_180_P_90_	311.11 ± 58.77a	147.50 ± 7.62c	51.90 ± 0.01abc	18.91 ± 0.42f	5,353.1 ± 1,126.45abcd
		N_90_P_45_	290.48 ± 17.17ab	141.00 ± 11.00c	51.38 ± 0.04abc	18.65 ± 0.26f	5,144.6 ± 387.28abcd
		N_0_P_0_	258.73 ± 31.71abc	137.41 ± 9.72c	55.07 ± 0.04abc	19.36 ± 0.81ef	4,808.7 ± 366.91cd
	Guiyu 9	N_180_P_90_	226.99 ± 23.49bc	177.65 ± 61.01bc	44.80 ± 0.07c	21.33 ± 1.60def	5,436.6 ± 723.10abcd
		N_90_P_45_	217.46 ± 35.10bc	141.81 ± 13.45c	49.10 ± 0.09bc	22.22 ± 2.37cde	5,081.4 ± 852.28bcd
		N_0_P_0_	190.48 ± 16.50c	147.47 ± 15.62c	50.84 ± 0.07abc	22.00 ± 1.03cde	4,350.9 ± 198.15d
	Y Liangyou 1	N_180_P_90_	255.56 ± 26.23abc	151.68 ± 7.80c	58.76 ± 0.01ab	26.41 ± 1.63ab	7,047.3 ± 410.20a
		N_90_P_45_	239.68 ± 30.60abc	155.58 ± 8.97c	51.79 ± 0.02abc	27.84 ± 0.39a	6,762.3 ± 694.85ab
		N_0_P_0_	246.03 ± 11.00abc	140.36 ± 18.53c	59.94 ± 0.01ab	26.8 ± 0.46ab	5,973.8 ± 335.22abcd
	Teyou 582	N_180_P_90_	188.89 ± 39.65c	243.15 ± 11.39a	53.96 ± 0.07abc	23.86 ± 0.29bcd	6,596.6 ± 941.22abc
		N_90_P_45_	179.37 ± 27.08cd	223.49 ± 14.31ab	54.33 ± 0.06abc	24.55 ± 0.24bc	6,824.4 ± 192.24ab
		N_0_P_0_	188.89 ± 19.25c	188.14 ± 8.51abc	62.56 ± 0.02a	23.89 ± 1.11bcd	5,370.5 ± 543.71abcd

**Note: **

Values in columns with different letters showed significant differences (*P* < 0.05). ± Values represent SE among the replications.

In the Liucheng test site, the effective panicle number of Baixiang 139 increased by 56.7% compared with Guiyu 9 (Tables 6 and 7). Furthermore, Baixiang 139 treated with N_180_P_90_ in Binyang had significantly more panicles (3.5802 million panicle ha^−1^) compared with other treatments. However, the panicle number of Teyou 582 at Beiliu was low (1.4445 million ear ha^−1^) under N_0_P_0_ treatment.

#### Grains panicle^−1^

The number of grains panicle^−1^ among different treatments was 28.6% and 16.7% higher in Liucheng compared with Binyang during the early and late seasons, respectively. Teyou 582 had a 49.5% higher number of grains per panicle compared with Baixiang 139 during the early season (Tables 6 and 7). In addition, Teyou 582 had considerably more (228.4 grains) total grains panicle^−1^ treated with N_90_P_45_ compared with treatments at Liucheng during the early season. Guiyu 9 treated with N_180_P_90_ had significantly more grains panicle^−1^ at Beiliu (113.84 grains) and Liucheng (243.15 grains) during the early and late seasons, respectively. The number of grains panicle^−1^ of Guiyu 9 was 37.7% higher compared with Baixiang 139 during the late season. However, Y Liangyou 1 at Binyang had a low number of grains panicle^−1^ (94 grains panicle^−1^) under the N_0_P_0_ treatment during the late season.

#### Seed setting rate

The seed setting rate was 3.1% higher in Liucheng than in Beiliu during the early season across fertilizer treatments (Tables 6 and 7). During the late season, the seed setting rate at Beiliu was 29.8% higher compared with Binyang, followed by Liucheng. Teyou 582 had a 34.5% higher seed setting rate than Guiyu 9 during the early season, and Y Liangyou 1 had a 19.1% higher seed setting rate than Teyou 582 during the late season. Teyou 582 treated with N_90_P_45_ and Y Liangyou 1 treated with N_180_P_90_ had seed setting rates that were 82.91% and 72.17% higher compared with other treatments at Beiliu in the early and late seasons, respectively. The lowest seed setting rate was recorded in Guiyu 9 (at Beiliu) and Teyou 582 (at Binyang) under N_90_P_45_ treatment during the early and late seasons, respectively.

#### Thousand-grain weight

TGW of Binyang between different treatments during the early season was 7.5% higher than that of Liucheng, and TGW of Beiliu between different treatments during the late season was 1.8% higher than that of Liucheng.Y Liangyou 1 had 44% higher TGW compared with Baixiang during both seasons (Tables 6 and 7). N_0_P_0_ and N_90_P_45_ increased the TGW of Y Liangyou 1 at Binyang (27.07 g) and Liucheng (27.84 g) during the early and late seasons, respectively. Lower TGW values (17.05 and 17.5 gm) were observed in Baixiang 139 at Liucheng and Beiliu during the early and late seasons, respectively, under N_0_P_0_.

#### Grain yield

The yield of Beiliu among different treatments during the early season was 27.5% higher compared with Liucheng (Tables 6 and 7). Compared with Guiyu 9, the yield of Y Liangyou 1 was increased by 32.3%. In Beiliu, the N_90_P_45_ treatment (6,611.7 kg ha^−1^) of Teyou 582 increased production compared with the other treatments. In Liucheng, the grain yield of Baixiang 139 was lower (3,482.3 kg ha^−1^) under N_0_P_0_ treatment.

### Relationships between DM accumulation, LAI, effective panicle number, total number of grains per panicle, and grain yield of rice

Rice yield is strongly related to yield components and growth attributes (Iqbal et al., 2020). In this study, the correlation analysis showed that DM (0.65**), LAI (0.6**), and effective panicle number (0.56*) were strongly positively correlated with grain yield (Table 8). Total grains panicle^−1^, seed setting rate, and TGW were moderately positively correlated with grain yield. Furthermore, effective panicle number was also strongly positively correlated with DM (0.83**) and LAI (0.6**). DM was significantly correlated with LAI (0.78**) and total grains panicle^−1^ (0.52*).

**Table 8:** Correlation between rice yield and yield components.

Index	Grain yield	LAI	DM	EP	TGPP	SSR
LAI	0.6**					
DM	0.65**	0.78**				
Effective panicles (EP)	0.56*	0.6**	0.83**			
Total grains panicle^−1^	0.35	0.44	0.52*	0.03		
Seed setting rate	0.41	0.36	0.3	0.25	−0.1	
TGW	0.13	0.07	−0.21	−0.04	−0.21	−0.4

**Note:**

* Significant correlation (*P* < 0.05).

** Extremely significant correlation (*P* < 0.01).

LAI, leaf area index; DM, dry matter; TGW, thousand grain weight; EP, Effective panicles

## Discussion

Nitrogen (N) and phosphorous (P) fertilizers are key to the “green revolution,” which has converted approximately half of the world’s land to agriculture (Melillo, 2012; Iqbal et al., 2021). Previously it is well reported that using synthetic fertilizers in conventional farming improved crop growth and yield. Increasing the rate of N and P fertilizer application has been reported the main strategy for increasing grain yield (Leghari et al., 2016; Ali et al., 2019; Iqbal et al., 2021). However, the overuse of N and P can lead to the eutrophication of water bodies, high nitrate content in water bodies, and deterioration of rice quality (Carpenter et al., 1998; Ullah et al., 2020). Furthermore, the consequences of N and P losses from paddy fields, such as through runoff and leaching, can span multiple organizational levels and scales in time and space and thus threaten critical ecosystem services (Fowler et al., 2013; Guignard et al., 2017; Ullah et al., 2020). Although previous work indicates that increasing P and N fertilization can increase crop growth, yield, and yield attributes, and few studies have examined the extent to which the levels of P and N fertilizers could be reduced at different sites and with different cultivars in southern China. Thus our studies provided the response of different cultivars to the reduction of fertilizer use in South China at three different experimental sites.

Leaf area index (LAI) and dry matter (DM) are directly associated with the grain yield of rice (Iqbal et al., 2019; Ali et al., 2020). Our results showed that the LAI was increased under moderate amounts of fertilization (N9_0_P_45_) of a hybrid cultivar (Guiyu 9) compared with conventional rice cultivars. Likewise, moderate fertilization (N_90_P_45_) led to higher DM of a hybrid variety (Teyou 582) at Beiliu. These increases can be explained by the characteristics of hybrid cultivars, which can use less N and P while maintaining LAI and DM in paddy fields. These cultivars require less N and P for their growth and development (Li et al., 2019). Our results are consistent with those of Ali et al. (2020), showing that proper N application can increase the number of tillers and LAI of rice and promote its growth but that the excessive application of N fertilizer increases the number of ineffective tillers and reduces the utilization of N (Xie et al., 2021). However, several studies have reported that reducing N and P fertilizer application reduces rice growth traits (Jiang et al., 2021; Murthy et al., 2015) and alters rice flowering days (Ye et al., 2019). Similar to our findings, another study reported that reducing N application rate increases rice grain yield and N use efficiency (Wei et al., 2021), although their study used dense planting techniques. In addition Zhang et al. (2014) documented that regulating fertilization rate such as reducing and postponing N doses could sustain plant growth and yield. In general, our results suggest that rice LAI can be enhanced with the use of hybrid cultivars treated with moderate amounts of fertilizer.

Furthermore, our results indicated that effective panicle number and grains per panicle were highest under N_180_P_90_ fertilization of Baixiang 139 and Guiyu 9, respectively. Seed setting rate, TGW, and grain yield were highest under moderate fertilization (N_90_P_45_) in Beiliu with the cultivar Teyou 582; furthermore, Y Liangyou 1 led to a higher grain yield compared with Guiyu 9 in Liucheng. Overall, the hybrid cultivar Teyou 582 increased the grain yield and yield attributes under reduced fertilization rates (N_90_P_45_); however, there were no significant differences in grain yield and yield attributes between moderate and high applications of N and P fertilizer. This result might stem from the properties of Teyou 582, a hybrid cultivar that can grow better and produce higher yields with less fertilizer. These results are consistent with those of (Ma et al., 2019) showing that optimal applications of N and P enhanced grain yield and quality by improving N uptake. Furthermore, similar to our finding reported that using low fertilizer rates improves rice yield with other agronomic techniques such as selection of verities (Ju et al., 2015), dense planting (Wei et al., 2021), coupling with organic manure (Iqbal et al., 2019) or combined with biochar amendment (Ali et al., 2020) whereas other studies (Wang et al., 2011) have found that moderate application rates of N lead to increases in nitrate content in the 0–60 cm soil layer, N uptake amount, grain yield, and apparent recovery fraction of applied fertilizer N in wheat (Cheng et al., 2020; Tadesse et al., 2020) also showed that moderate amounts of N and P can enhance grain yield compared with higher amounts of N and P in wheat and rice.

## Conclusion

Based on our results, we concluded that rice grain yield can be increased under lower fertilization rate using hybrid cultivars. Our results suggested that low fertilization rates of 90 kg N ha^−1^ and 45 kg P ha^−1^ can increase grain yield and yield attributes in hybrid cultivars. However, hybrid and conventional cultivars of rice lead to similar yields under higher fertilization rates. Thus, the use of rice hybrid cultivars under a reduced fertilization rate might allow higher grain yields with a lower environmental cost. In order to confirm the potential of these cultivars, further studies are needed to determine the quality, initial photosynthetic rates, root attributes, and chlorophyll fluorescence of these cultivars under reduced fertilization rates.

## Supplemental Information

10.7717/peerj.11668/supp-1 Supplemental Information 1 Leaf Area RAW data.

10.7717/peerj.11668/supp-2 Supplemental Information 2 Dry matter RAW data.

10.7717/peerj.11668/supp-3 Supplemental Information 3 Yield and Yield component RAW Data.

## Additional Information and Declarations

### Field Study Permissions

The following information was supplied relating to field study approvals (i.e., approving body and any reference numbers): The field used in these experiments belong to the projects of our respected supervisor and corresponding author Professor Jiang Ligeng.

### Data Availability

The following information was supplied regarding data availability: Raw data are available as Supplemental Files.

## References

[ref-1] Acevedo-Siaca LG, Coe R, Wang Y, Kromdijk J, Quick WP, Long SP (2020). Variation in photosynthetic induction between rice accessions and its potential for improving productivity. New Phytologist.

[ref-2] Ali I, Khan AA, Khan A, Asim M, Ali I, Zib B, Khan I, Rab A, Sadiq G, Ahmad N, Iqbal B (2019). Humic acid and nitrogen levels optimizing productivity of green gram (Vigna radiate L.). Russian Agricultural Sciences.

[ref-3] Ali I, Ullah S, He L, Zhao Q, Iqbal A, Wei S, Shah T, Ali N, Bo Y, Adnan M, Jiang L (2020). Combined application of biochar and nitrogen fertilizer improves rice yield, microbial activity and N-metabolism in a pot experiment. PeerJ.

[ref-4] Angus JF, Ohnishi M, Horie T, Williams RL (1994). A preliminary study to predict net nitrogen mineralisation in a flooded rice soil using anaerobic incubation. Australian Journal of Experimental Agriculture.

[ref-5] Bai Z, Ma L, Jin S, Ma W, Velthof GL, Oenema O, Liu L, Chadwick D, Zhang F (2016). Nitrogen, phosphorus, and potassium flows through the manure management chain in China. Environmental Science & Technology.

[ref-6] Beckinghausen A, Odlare M, Thorin E, Schwede S (2020). From removal to recovery: an evaluation of nitrogen recovery techniques from wastewater. Applied Energy.

[ref-7] Cai CZ, Liang Y, Wan HT (2010). Analyses of rice yield in China based on the projection of yield potential. Chinese Agricultural Science Bulletin.

[ref-8] Cambardella CA, Gajda AM, Doran JW, Wienhold BJ, Kettler TA, Lal R, Lal R, Kimble JM, Follett RF, Stewart BA (2001). Estimation of particulate and total organic matter by weight loss-on-ignition. Assessment Methods for Soil Carbon.

[ref-9] Carpenter SR, Caraco NF, Correll DL, Howarth RW, Sharpley AN, Smith VH (1998). Nonpoint pollution of surface waters with phosphorus and nitrogen. Ecological Applications.

[ref-11] Cheng Y, Bao Y, Chen X, Yao Q, Wang C, Chai S, Zeng J, Fan X, Kang H, Sha L, Zhang H (2020). Different nitrogen forms differentially affect Cd uptake and accumulation in dwarf Polish wheat (Triticum polonicum L.) seedlings. Journal of Hazardous Materials.

[ref-12] Curtin D, Wright C, Beare M, McCallum FJSSSOAJ (2006). Hot water-extractable nitrogen as an indicator of soil nitrogen availability. Soil Science Society of America Journal.

[ref-51] Dhillon J, Torres G, Driver E, Figueiredo B, Raun WR (2017). World phosphorus use efficiency in cereal crops. Agronomy Journal.

[ref-13] Fowler D, Pyle JA, Raven JA, Sutton MA (2013). The global nitrogen cycle in the twenty-first century. Philosophical Transactions of the Royal Society B.

[ref-14] Gao MF, Qiu JJ, Liu SC, Liu HB, Wang LG, Pang HC (2014). Status and trends of agricultural diffuse pollution research based on bibliometrics. Scientia Agricultura Sinica.

[ref-15] Guignard MS, Leitch AR, Acquisti C, Eizaguirre C, Elser JJ, Hessen DO, Jeyasingh PD, Neiman M, Richardson AE, Soltis PS, Soltis DE (2017). Impacts of nitrogen and phosphorus: from genomes to natural ecosystems and agriculture. Frontiers in Ecology and Evolution.

[ref-16] Heffer P, Gruère A, Roberts T (2014). Assessment of fertilizer use by crop at the global level, International Fertilizer Association (IFA) and International Plant Nutrition Institute (IPNI). https://www.fertilizer.org/images/Library_Downloads/2017_IFA_AgCom_17_134%20rev_FUBC%20assessment%202014.pdf.

[ref-18] Hirzel J, Paredes M, Becerra V, Donoso G (2020). Response of direct seeded rice to increasing rates of nitrogen, phosphorus, and potassium in two paddy rice soils. Chilean Journal of Agricultural.

[ref-19] Iqbal A, He L, Khan A, Wei S, Akhtar K, Ali I, Ullah S, Munsif F, Zhao Q, Jiang L (2019). Organic manure coupled with inorganic fertilizer: an approach for the sustainable production of rice by improving soil properties and nitrogen use efficiency. Agronomy.

[ref-50] Iqbal A, He L, Ali I, Ullah S, Khan A, Khan A, Akhtar K, Wei S, Zhao Q, Zhang J, Jiang L (2020). Manure combined with chemical fertilizer increases rice productivity by improving soil health, post-anthesis biomass yield, and nitrogen metabolism. PLOS ONE.

[ref-20] Iqbal A, Xie H, He L, Ahmad S, Hussain I, Raza H, Khan A, Wei S, Quan Z, Wu K, Ali I (2021). Partial substitution of organic nitrogen with synthetic nitrogen enhances rice yield, grain starch metabolism and related genes expression under the dual cropping system. Saudi Journal of Biological Sciences.

[ref-21] Jackson ML (1956). Soil chemical analysis-Advanced course, published by the author.

[ref-22] Jiang B, Jianlin SH, Minghong SU, Yajun HU, Jiang W, Juan WA, Yong LI, Jinshui WU (2021). Soil phosphorus availability and rice phosphorus uptake in paddy fields under various agronomic practices. Pedosphere.

[ref-23] Ju C, Buresh RJ, Wang Z, Zhang H, Liu L, Yang J, Zhang J (2015). Root and shoot traits for rice varieties with higher grain yield and higher nitrogen use efficiency at lower nitrogen rates application. Field Crops Research.

[ref-24] Leaf AL (1958). Determination of available potassium in soils of forest plantations. Soil Science Society of America Journal.

[ref-25] Leghari SJ, Wahocho NA, Laghari GM, HafeezLaghari A, MustafaBhabhan G, HussainTalpur K, Bhutto TA, Wahocho SA, Lashari AA (2016). Role of nitrogen for plant growth and development: a review. Advances in Environmental Biology.

[ref-49] Li F, Dai X, Wang L, Xia X, Wei S, Liang H, Jiang L (2019). Evaluation and classification of low fertilizer tolerance of main rice varieties in Guangxi [J]. Guangdong Agricultural Sciences.

[ref-26] Li LI, Xi-zhou ZH, Ting-xuan LI, Hai-ying YU, Lin JI, Guang-deng CH (2014). Characteristics of phosphorus uptake and use efficiency of rice with high yield and high phosphorus use efficiency. Yingyong Shengtai Xuebao.

[ref-27] Lin Z, Qin P, Zhang X, Fu C, Deng H, Fu X, Huang Z, Jiang S, Li C, Tang X, Wang X (2020). Divergent selection and genetic introgression shape the genome landscape of heterosis in hybrid rice. Proceedings of the National Academy of Sciences of the United States of America.

[ref-28] Lu C, Tian H (2017). Global nitrogen and phosphorus fertilizer use for agriculture production in the past half century: shifted hot spots and nutrient imbalance. Earth System Science Data.

[ref-29] Ma G, Liu W, Li S, Zhang P, Wang C, Lu H, Wang L, Xie Y, Ma D, Kang G (2019). Determining the optimal N input to improve grain yield and quality in winter wheat with reduced apparent N loss in the North China Plain. Frontiers in Plant Science.

[ref-30] Melillo ED (2012). The first green revolution: debt peonage and the making of the nitrogen fertilizer trade, 1840–1930. The American Historical Review.

[ref-31] Murthy KM, Rao AU, Vijay D, Sridhar TV (2015). Effect of levels of nitrogen, phosphorus and potassium on performance of rice. Indian Journal of Agricultural Research.

[ref-32] Ohyma T, Ito M, Kobayashi K, Araki S, Yasuyoshi S, Sasaki O, Ikarashi T (1991). Analytical procedures of N, P, K contents in plant and manure materials using H2SO4-H2O2 Kjeldahl digestion method. Bulletin of the Faculty of Agriculture, Niigata University.

[ref-33] Olsen SR (1964). Estimation of available phosphorus in soils by extraction with sodium bicarbonate.

[ref-34] Rich CI, Black WR (1994). Pottasium exchange as affected by cation size, pH, and mineral structure. Soil Science.

[ref-36] Steel RGD, Torrie JH, Dickey D (1996). Principles and procedures of statistics.

[ref-37] Tadesse T, Tadesse Z, Asega H, Abaychew D (2020). Optimum nitrogen and phosphors fertilizer rates for upland rice production in North Western Ethiopia. Journal of Agriculture and Environmental Sciences.

[ref-38] Tao R, Liang Y, Wakelin SA, Chu G (2015). Supplementing chemical fertilizer with an organic component increases soil biological function and quality. Applied Soil Ecology.

[ref-39] Ullah S, Liang H, Ali I, Zhao Q, Iqbal A, Wei S, Shah T, Yan B, Jiang L (2020). Biochar coupled with contrasting nitrogen sources mediated changes in carbon and nitrogen pools, microbial and enzymatic activity in paddy soil. Journal of Saudi Chemical Society.

[ref-40] Ullah S, Zhao Q, Wu K, Ali I, Liang H, Iqbal A, Wei S, Cheng F, Ahmad S, Jiang L, Gillani SW (2021). Biochar application to rice with 15N-labelled fertilizers, enhanced leaf nitrogen concentration and assimilation by improving morpho-physiological traits and soil quality. Saudi Journal of Biological Sciences.

[ref-41] Wang D, Xu Z, Zhao J, Wang Y, Yu Z (2011). Excessive nitrogen application decreases grain yield and increases nitrogen loss in a wheat-soil system. Acta Agriculturae Scandinavica, Section B-Soil & Plant Science.

[ref-42] Wehmeyer H, de Guia AH, Connor M (2020). Reduction of fertilizer use in South China—impacts and implications on smallholder rice farmers. Sustainability.

[ref-43] Wei H, Meng T, Ge J, Zhang X, Shi T, Ding E, Lu Y, Li X, Tao Y, Chen Y, Li M (2021). Reduced nitrogen application rate with dense planting improves rice grain yield and nitrogen use efficiency: a case study in east China. The Crop Journal.

[ref-52] Xie H, Wu K, Iqbal A, Ali I, He L, Ullah S, Wei S, Zhao Q, Wu X, Huang Q, Jiang L (2021). Synthetic nitrogen coupled with seaweed extract and microbial inoculants improves rice (*Oryza sativa* L.) production under a dual cropping system. Italian Journal of Agronomy.

[ref-44] Ye T, Li Y, Zhang J, Hou W, Zhou W, Lu J, Xing Y, Li X (2019). Nitrogen, phosphorus, and potassium fertilization affects the flowering time of rice (Oryza sativa L.). Global Ecology and Conservation.

[ref-45] Zhang A, Liu R, Gao J, Yang S, Chen Z (2014). Regulating N application for rice yield and sustainable eco-agro development in the upper reaches of Yellow River Basin, China. The Scientific World Journal.

[ref-46] Zhao Q, Hao X, Ali I, Iqbal A, Ullah S, Huang M, Kong F, Li T, Xuan Y, Li F, Yan B (2020). Characterization and grouping of all primary branches at various positions on a rice panicle based on grain growth dynamics. Agronomy.

[ref-47] Zhou K, Barjenbruch M, Kabbe C, Inial G, Remy C (2017a). Phosphorus recovery from municipal and fertilizer wastewater: China’s potential and perspective. Journal of Environmental Sciences.

[ref-53] Zhou J, Jiang X, Wei D, Zhao B, Ma M, Chen S, Cao F, Shen D, Guan D, Li J (2017b). Consistent effects of nitrogen fertilization on soil bacterial communities in black soils for two crop seasons in China. Scientific Reports.

[ref-48] Zörb C, Grover C, Steinfurth D, Mühling KH (2010). Quantitative proteome analysis of wheat gluten as influenced by N and S nutrition. Plant and Soil.

